# The grapevine (*Vitis vinifera*) LysM receptor kinases VvLYK1‐1 and VvLYK1‐2 mediate chitooligosaccharide‐triggered immunity

**DOI:** 10.1111/pbi.13017

**Published:** 2018-10-22

**Authors:** Daphnée Brulé, Clizia Villano, Laura J. Davies, Lucie Trdá, Justine Claverie, Marie‐Claire Héloir, Annick Chiltz, Marielle Adrian, Benoît Darblade, Pablo Tornero, Lena Stransfeld, Freddy Boutrot, Cyril Zipfel, Ian B. Dry, Benoit Poinssot

**Affiliations:** ^1^ Agroécologie Agrosup Dijon INRA Université Bourgogne Franche‐Comté CNRS ERL 6003 Dijon France; ^2^ University of Naples Federico II Portici Naples Italy; ^3^ Commonwealth Scientific and Industrial Research Organisation (CSIRO) Adelaide SA Australia; ^4^ Elicityl Crolles France; ^5^ Instituto de Biología Molecular y Celular de Plantas Universitat Politècnica de València Consejo Superior de Investigaciones Científicas Valencia Spain; ^6^ The Sainsbury Laboratory Norwich Research Park Norwich UK

**Keywords:** pathogen‐associated molecular pattern, pattern recognition receptor, *Vitis vinifera*, immune responses, *Erysiphe necator*, resistance

## Abstract

Chitin, a major component of fungal cell walls, is a well‐known pathogen‐associated molecular pattern (PAMP) that triggers defense responses in several mammal and plant species. Here, we show that two chitooligosaccharides, chitin and chitosan, act as PAMPs in grapevine (*Vitis vinifera*) as they elicit immune signalling events, defense gene expression and resistance against fungal diseases. To identify their cognate receptors, the grapevine family of LysM receptor kinases (LysM‐RKs) was annotated and their gene expression profiles were characterized. Phylogenetic analysis clearly distinguished three *V. vinifera* LysM‐RKs (VvLYKs) located in the same clade as the *Arabidopsis* CHITIN ELICITOR RECEPTOR KINASE1 (AtCERK1), which mediates chitin‐induced immune responses. The *Arabidopsis* mutant *Atcerk1*, impaired in chitin perception, was transformed with these three putative orthologous genes encoding VvLYK1‐1, ‐2, or ‐3 to determine if they would complement the loss of AtCERK1 function. Our results provide evidence that VvLYK1‐1 and VvLYK1‐2, but not VvLYK1‐3, functionally complement the *Atcerk1* mutant by restoring chitooligosaccharide‐induced MAPK activation and immune gene expression. Moreover, expression of *VvLYK1‐1* in *Atcerk1* restored penetration resistance to the non‐adapted grapevine powdery mildew (*Erysiphe necator*). On the whole, our results indicate that the grapevine VvLYK1‐1 and VvLYK1‐2 participate in chitin‐ and chitosan‐triggered immunity and that VvLYK1‐1 plays an important role in basal resistance against *E. necator*.

## Introduction

Plants are constantly exposed to potentially pathogenic microbes such as bacteria, fungi, oomycetes or viruses. However, plants have developed effective immune systems triggering various defense reactions against invading pathogens upon the perception of pathogen‐associated molecular patterns (PAMPs; Dodds and Rathjen, 2010). The recognition of these conserved microbial signatures is mediated by pattern recognition receptors (PRRs), which also detect plant endogenous molecules released by hydrolytic enzymes during interaction with the pathogen, and called damage‐associated molecular patterns (DAMPs; Boller and Felix, 2009; Boutrot and Zipfel, 2017). PRRs have a characteristic structure defined by the presence of a ligand‐binding ectodomain, a single transmembrane domain and, for some of them, an intracellular kinase domain. The structure of the ectodomain determines binding specificity: PRRs containing a leucine‐rich repeat ectodomain mostly bind peptides, such as flagellin or elongation factor Tu (EF‐Tu) from bacteria, whilst lysine motif (LysM)‐containing PRRs preferentially bind carbohydrates, such as chitin or peptidoglycans, from fungi and bacteria, respectively (Boutrot and Zipfel, 2017; Trdá *et al*., 2015). PAMP perception by PRRs leads to PAMP‐triggered immunity (PTI), which is characterized by a wide range of defense responses including the production of reactive oxygen species (ROS), calcium influx, mitogen‐ activated protein kinase (MAPK) phosphorylation and expression of defense‐related genes (Yu *et al*., 2017).

Several distinct microbial patterns are composed from *N*‐acetylglucosamine (GlcNAc) residues, including fungal chitin or bacterial peptidoglycan (PGN) present in microbial cell walls (Gust *et al*., 2012). Chitin, and its derivatives, are representative PAMPs from fungal cell walls known to induce immune responses in both monocots and dicots, indicating the presence of a conserved mechanism to perceive these chitooligosaccharides in a wide range of plant species (Shinya *et al*., 2015). In plants, chitin elicits a variety of defense responses including the activation of the phenylpropanoid pathway and production of pathogenesis‐related (PR) proteins such as peroxidases, chitinases, or thaumatin‐like proteins (Boller and Felix, 2009; Kaku *et al*., 2006; Miya *et al*., 2007). Chitosan, a deacetylated derivative of chitin, is also a potent elicitor of plant immunity (Aziz *et al*., 2006; Povero *et al*., 2011). In grapevine, chitosan elicits phytoalexin production, chitinase and glucanase activities leading to resistance against *Botrytis cinerea* and *Plasmopara viticola*, the causal agents of grey mould and downy mildew, respectively (Aziz *et al*., 2006).

The mechanism of chitin perception and signalling in plant cells was first characterized in rice with the identification of the chitin‐elicitor binding protein, CEBiP (Kaku *et al*., 2006), which contains three extracellular LysM motifs and is anchored to the plasma membrane via a glycosylphosphatidylinositol (GPI)‐anchor (Gong *et al*., 2017). Chitin perception in rice triggers the formation of a heterodimer complex between OsCEBiP and OsCERK1, a protein which contains an intracellular kinase domain required for signal transduction. Thus, two LysM proteins are required for chitin perception and signalling in rice (Hayafune *et al*., 2014; Shimizu *et al*., 2010). In *Arabidopsis thaliana*, AtCERK1/LYK1, a homolog of OsCERK1, has been shown to play a crucial role in both chitin signalling (Miya *et al*., 2007; Wan *et al*., 2008) and bacterial PGN perception (Gimenez‐Ibanez *et al*., 2009; Willmann *et al*., 2011). Homodimers of AtCERK1/LYK1 were shown to directly bind long chain chitin oligomers (Liu *et al*., 2012). However, more recent data suggest that other members of the LysM‐RK gene family in *Arabidopsis* may also be involved in chitin perception. For example, Cao *et al*. (2014) proposed that AtLYK5 (and/or AtLYK4), which have inactive kinase domains, may be the primary receptors for chitin, and that chitin perception may result in the formation of an AtLYK5‐AtLYK1 heterotetramer, triggering intracellular signal transduction.

The majority of commercially grown grapevine cultivars are derived from the species *Vitis vinifera*, which is highly susceptible to cryptogamic diseases, such as downy mildew (*Plasmopara viticola*), grey mould (*Botrytis cinerea*) and powdery mildew (*Erysiphe necator*). These two last pathogens are ascomycete fungi containing chitooligosaccharides in their cell walls. These diseases cause significant losses to viticultural production and control of these pathogens is heavily dependent on frequent fungicide application. The level of fungicide application has serious economic, environmental and potential health implications and has driven research efforts into alternative strategies (Trouvelot *et al*., 2014; Walters *et al*., 2013). Among them is the generation of new resistant varieties by introgression of downy and powdery mildew resistance (*R*) genes from wild North American grapevine species (Qiu *et al*., 2015). However, whilst *R*‐gene triggered resistance is very effective at controlling pathogens, widespread use of *R*‐genes may impose a selection pressure on parasites to evolve and evade R protein recognition, thereby compromising the durability of this control strategy (Jones and Dangl, 2006). Thus, characterization of new PRRs in a given plant species by identifying their cognate PAMPs and understanding their involvement in disease resistance may provide more durable and broad‐spectrum immunity (Piquerez *et al*., 2014), notably by promoting a PTI‐based crop protection (Boutrot and Zipfel, 2017; Wiesel *et al*., 2014).

In this study, we have investigated whether two chitooligosaccharides, chitin and chitosan, are active PAMPs in grapevine. We also report on the functional characterization of members of the *VvLYK* gene family, with particular focus on three orthologs of *AtCERK1/LYK1* and *OsCERK1*, designated *VvLYK1‐1*,* VvLYK1‐2* and *VvLYK1‐3*. By functional complementation of the *Arabidopsis Atcerk1* mutant, we demonstrate that *VvLYK1‐1* and *VvLYK1‐2* are involved in the chitooligosaccharide‐induced immune responses in *V. vinifera*. Moreover, *VvLYK1‐1* was demonstrated to confer basal resistance against the grapevine powdery mildew *E. necator* when expressed in *A. thaliana*.

## Results

### Chitooligosaccharides trigger immune responses and induced resistance in grapevine

Chitooligosaccharides with a degree of polymerization (DP) ranging from 6 to 8 (hexamer to octamer) are the most effective at triggering ROS production and defense gene expression in rice and *Arabidopsis*, respectively (Miya *et al*., 2007; Petutschnig *et al*., 2010). In grapevine, chito‐oligosaccharides with a MW of 1500 (i.e. DP6) were shown to be the most effective at triggering phytoalexin production and expression of chitinase and glucanase, compared to chitooligosaccharides with a MW of 3000 and 10 000 (i.e. DP13–45) (Aziz *et al*., 2006). In this study, chitooligomers with a DP of 6 were used to test if their perception by grapevine triggers immune responses similar to that commonly observed in *Arabidopsis* or rice. To also investigate the importance of the degree of acetylation (DA), the early signalling events and defense gene expression induced by chitin hexamer (DA 99.9% and DP 6) or deacetylated chitosan hexamer (DA 0.1% and DP 6) were characterized in *V. vinifera* cell suspensions.

Contrary with what has been previously observed in *Arabidopsis* (Albert *et al*., 2006; Miya *et al*., 2007), chitin DP6 did not induce any oxidative burst in grapevine cells (Figure S1) whereas flg22 triggered the expected positive response (Trdá *et al*., 2014). Similarly, the fully deacetyled chitosan DP6 did not elicit any H_2_O_2_ production in grapevine cell suspension (Figure S1).

**Figure 1:**
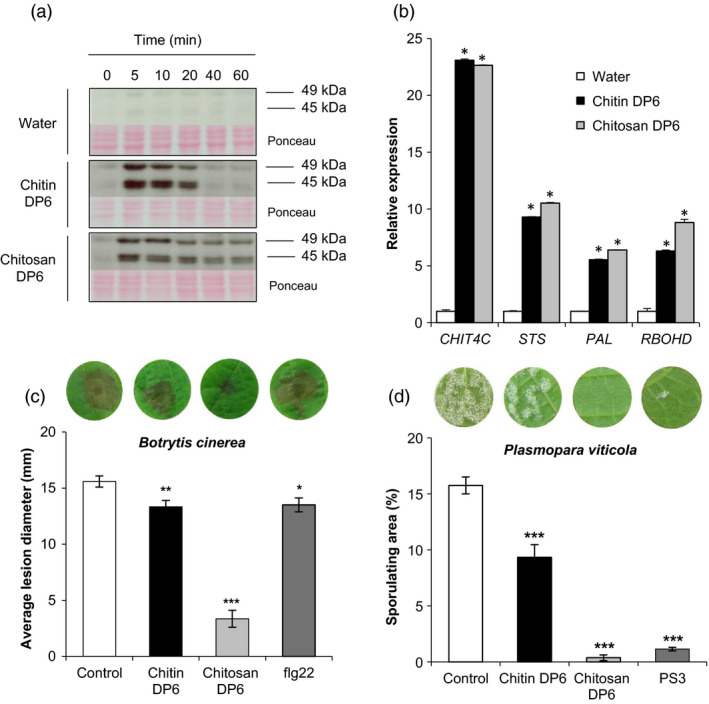
Chitin and chitosan induced defense responses and resistance to pathogens in grapevine. (a) Activation kinetics of two mitogen‐activated protein kinases (MAPKs) detected by immunoblotting with an antibody raised against the human phosphorylated extracellular regulated protein kinase 1/2 (α‐pERK1/2) in grapevine cells treated with chitin DP6 (100 μg/mL), chitosan DP6 (100 μg/mL) or water (negative control). Homogeneous loading was checked by Ponceau red staining. (b) Expression of defense genes encoding an acidic chitinase (*Chit4C*), a stilbene synthase (*STS*), a phenylalanine ammonia lyase (*PAL*) and a respiratory burst oxidase homolog D (*RbohD)* measured by quantitative polymerase chain reaction (qPCR) 1 h post‐treatment with chitin DP6 (100 μg/mL), chitosan DP6 (100 μg/mL) or water. Values represent the mean of triplicate data ±SE (*n* = 3) from one experiment out of three and data were normalized by the housekeeping gene *EF1*α and compared with water (negative control), set as 1. Asterisks (*) indicate statistically significant differences between water and chitooligosaccharide treatment, using an unpaired heteroscedastic Student's *t* test (*P* < 0.05). (c) Development of *B. cinerea* at 3 days post‐inoculation (dpi) on grapevine leaf discs treated 48 h before with chitin DP6 (1 mg/mL), chitosan DP6 (1 mg/mL) or flg22 (10 μm) previously solubilized in Dehscofix 0.1% and compared with control (adjuvant : Dehscofix 0.1%). Values represent the mean of lesion diameters ±SE (*n* ≥ 36 discs from three different plants) from one representative experiment out of three. (d) Sporulation caused by *P. viticola* at 8 dpi on grapevine leaf discs treated 48 h before inoculation with chitin DP6 (100 μg/mL), chitosan DP6 (100 μg/mL) or 2.5 mg/mL sulphated laminarin (PS3) previously solubilized in Dehscofix 0.1% and compared with control (adjuvant : Dehscofix 0.1%). Sporulating leaf area was evaluated by image analysis Visilog 6.9 software (Kim Khiook *et al*., 2013). Values represent the mean of percentage of sporulating area ±SE (*n* = 30 discs from three different plants) from one representative experiment out of three. Asterisks indicate a statistically significant difference between control and the elicitor treatment (Student's *t*‐test; *, *P* < 0.05, **, *P* < 0.01, ***, *P* < 0.001). A representative leaf disc for each treatment is shown. Similar results were obtained in at least three independent experiments.

However, chitin DP6 induced a rapid and transient phosphorylation of two MAPKs with relative molecular masses of 45 and 49 kDa, which was not observed in water‐treated control cells (Figure 1a). Interestingly, chitosan DP6 also activated the phosphorylation of these two MAPKs but for a longer period (Figure 1a). In parallel, treatment of grapevine cells with unpurified crab shell chitin NA‐COS‐Y, previously used to elicit ROS production and defense gene expression in *Brassica* species (Lloyd *et al*., 2014), was also shown to activate these two MAPKs (Figure S2).

In response to chitooligosaccharide treatment, the expression of defense genes known to be induced by different PAMPs in grapevine (Aziz *et al*., 2003; Dubreuil‐Maurizi *et al*., 2010; Poinssot *et al*., 2003; Trdá *et al*., 2014) was examined by qPCR. One hour post‐treatment (hpt), both chitin DP6 and chitosan DP6 markedly induced the expression of four selected grapevine defense genes (Figure 1b) encoding an acidic chitinase (*CHIT4C*), a stilbene synthase (*STS*), a phenylalanine ammonia lyase (*PAL*) and a respiratory burst oxidase homolog D (*RBOHD*).

To further characterize the immune responses triggered by chitooligosaccharides, we also investigated the efficacy of chitin‐ and chitosan‐induced resistance in grapevine. Leaf discs were treated with chitin DP6 and chitosan DP6 for 48 h prior to inoculation with either the necrotrophic fungus *B. cinerea* or with the biotrophic oomycete *P. viticola*. Chitin treatment induced a low but significant resistance against these pathogens (Figure 1c, d), whilst chitosan treatment significantly reduced *B. cinerea* lesion diameter and *P. viticola* sporulation (Figure 1c, d). Indeed, the reduced susceptibility to *P. viticola* infection, triggered by chitosan, was comparable to that obtained by pretreatment with the β‐1,3‐glucan sulphated laminarin (PS3), a potent resistance inducer in grapevine (Gauthier *et al*., 2014).

### Phylogenetic analysis and characterization of grapevine LysM‐RKs (VvLYKs)

**Figure 2:**
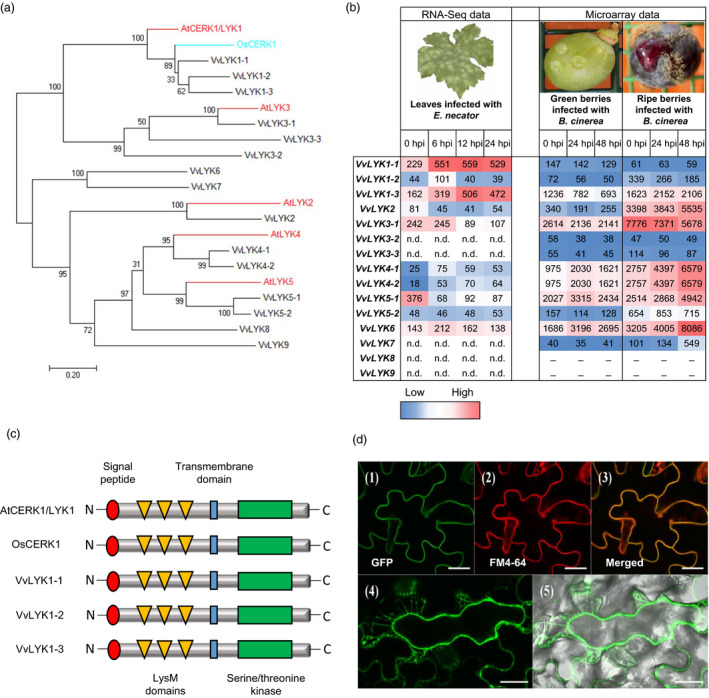
Phylogenetic analysis and characterization of grapevine LysM‐RKs (VvLYKs). (a) Maximum‐likelihood phylogenetic tree drawn with MEGA 7 (Kumar et al., 2016) showing the relationship between the Arabidopsis proteins AtCERK1/LYK1 and AtLYK2‐5 (red), the rice OsCERK1 (blue) and the most similar protein sequences of *Vitis vinifera* (black). Sequences used for the phylogenetic analysis were: AtCERK1/LYK1 (NP_566689), AtLYK2 (OAP05017), AtLYK3 (NP_175606), AtLYK4 (NP_179957), AtLYK5 (NP_180916), OsCERK1 (A0A0P0XII1), VvLYK1‐1 (XP_010657225), VvLYK1‐2 (XP_010655366), VvLYK1‐3 (XP_010655365), VvLYK2 (XP_019080819), VvLYK3‐1 (XP_002283628), VvLYK3‐2 (XP_019074828), VvLYK3‐3 (XP_002272814), VvLYK4‐1 (XP_002269408), VvLYK4‐2 (XP_010649202), VvLYK5‐1 (XP_002277331), VvLYK5‐2 (MF177034), VvLYK6 (XP_002280070), VvLYK7 (XP_002269472), VvLYK8 (XP_002281880) and VvLYK9 (XP_002276830). (b) *VvLYK* expression profiles during *E. necator* or *B. cinerea* infection. Results are expressed as Relative Expression Values. Colour range has been made independently from RNA‐Seq or microarray data. (n.d. = no full length transcript detected in RNA Seq; _ = no specific probe available in microarray). (c) Schematic structure of AtCERK1/LYK1, OsCERK1, VvLYK1‐1, VvLYK1‐2 and VvLYK1‐3 based on the multiple alignment realized with T‐coffee (Figure S2). (d) Subcellular localization of VvLYK1‐1‐GFP in the line *Atcerk1/p35S::VvLYK1‐1‐GFP*. Leaves of *Arabidopsis thaliana* expressing VvLYK1‐1‐GFP were incubated with the plasma membrane dye FM4‐64. Confocal microscopy imaging revealed the green GFP‐tagged VvLYK1‐1 (1), the red FM4‐64 labelled plasma membrane (2) and the co‐localization of both probes in *Arabidopsis* leaves (3). (4) NaCl (1M) induced plasmolysis and confocal microscopy imaging revealed that VvLYK1‐1‐GFP fluorescence followed the plasma membrane shrinking (5). Bars, 20 μm.

The results of Figure 1 demonstrate that grapevine cells are capable of detecting chitooligosaccharides, suggesting the presence of a perception system. To identify the CERK1/LYK1 ortholog(s) in grapevine, genes encoding LysM‐RKs were identified from the reference genome of Vitis vinifera cv. Pinot Noir PN40024 (Jaillon *et al*., 2007). A previous annotation of the VvLYK family based on the 8x grapevine genome predicted 12 gene family members (Zhang *et al*., 2009). However, our re‐annotation of the *VvLYK* gene family, based on the most recent version of the 12x genome, predicts the presence of 15 putative genes encoding VvLYK proteins in the *V. vinifera* genome (Table S1). A maximum‐likelihood phylogenetic tree indicated that of these 15 LysM‐RKs, three grapevine proteins are located in the same clade as the *Arabidopsis* AtCERK1/LYK1 and the rice ortholog OsCERK1 (Figure 2a), proteins that have been shown to be involved in chitin perception/signalling. These proteins, designated as VvLYK1‐1, VvLYK1‐2 and VvLYK1‐3, share 60%, 57% and 56% amino acid identity with AtCERK1/LYK1, respectively (Table S2). VvLYK1‐1 and VvLYK1‐2 also show the highest percentage of amino acid identity with the rice chitin co‐receptor OsCERK1 (Table S2).

The expression profile of each putative *VvLYK* gene was analysed using RNA‐Seq and microarray data obtained from time course infection experiments of leaves and berries with *E. necator* and *B. cinerea* (Kelloniemi *et al*., 2015), respectively. In response to inoculation with the fungal pathogen *E. necator*, only *VvLYK1‐1* and *VvLYK1‐3* were clearly up‐regulated across the entire 24 h period, while *VvLYK1‐2* and *VvLYK6* were transiently induced at 6 hpi (Figure 2b). During *B. cinerea* infection, in the clade of VvLYK1s, only *VvLYK1‐3* was slightly induced in ripe susceptible berries (Figure 2b). Interestingly, *VvLYK4‐1/2* (detected using the same Nimblegen probe), *VvLYK5‐1* and *VvLYK6* were strongly up‐regulated in berries during infection by *B. cinerea* (Figure 2b). *VvLYK2* expression is also much higher in ripe berries than green berries, suggesting that it could have an as yet unknown function during grape berry ripening. *VvLYK3‐1* appeared to be repressed during the infection by both pathogens. Of note, we found *VvLYK3‐2*,* VvLYK3‐3*,* VvLYK7*,* VvLYK8* and *VvLYK9* to only be expressed at very low levels or were undetectable in the tissues examined (Figure 2b). However, we cannot rule out the possibility that these genes are expressed at detectable levels in other tissues, such as roots or flowers, or in response to other biotic stresses.

As AtCERK1 and OsCERK1 are the key components that mediate chitin‐triggered signalling in *Arabidopsis* (Miya *et al*., 2007; Wan *et al*., 2008) and rice (Hayafune *et al*., 2014; Shimizu *et al*., 2010), we undertook further analysis of the three putative grapevine orthologs *VvLYK1‐1*,* VvLYK1‐2* and *VvLYK1‐3*. Sequencing of the cloned full‐length coding sequences (CDS) from *V. vinifera* cv Cabernet Sauvignon revealed that genes *VvLYK1‐1*,* ‐2* or *‐3* consist of open‐reading frames of 1845, 1878 and 1866 bp, respectively (Table S1). All three VvLYK1 proteins contain a similar domain structure with a signal peptide, three extracellular LysM motifs, a single transmembrane domain and a RD‐type intracellular kinase domain (Figure 2c and Figure S3). Interestingly, the amino acids E110 and E114, shown to be involved in the binding of the N‐acetyl moieties of (GlcNAc)_5_ in AtCERK1/LYK1 (Liu *et al*., 2012) are mutated in the three VvLYK1 proteins (Figure S3). All three VvLYK1 protein sequences share a high degree of identity (Figure S3) and the kinase domains of VvLYK1‐1 and VvLYK1‐2 possess the highest identity with the kinase domains of AtCERK1/LYK1 and OsCERK1 (Figure S3, Table S2).

All three VvLYK1 proteins have a predicted N‐terminal signal peptide (Figure S3). Confocal analysis of the *Atcerk1* mutant expressing a *VvLYK1‐1‐GFP* fusion expression construct showed a GFP signal co‐localized with the red fluorescence of the plasma membrane‐specific probe FM4‐64 (Brandizzi *et al*., 2004) (Figure 2d). Furthermore, when plasmolysis was triggered by the addition of 1 m NaCl, VvLYK1‐1‐GFP fluorescence followed the movement of the plasma membrane away from the plant cell wall (Figure 2d). Both observations are consistent with VvLYK1‐1 being localized to the plasma membrane.

### VvLYK1‐1 restores chitin‐induced MAPK activation and *FRK1* expression in the *Atcerk1* mutant

**Figure 3:**
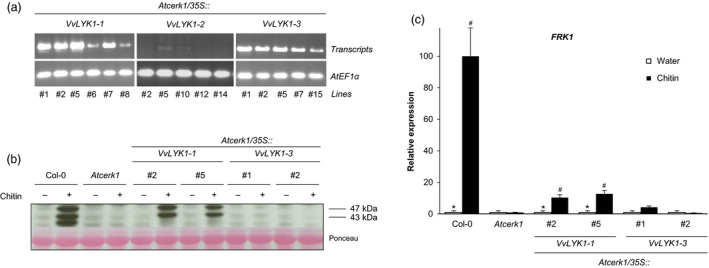
VvLYK1‐1 restores chitin‐induced immune responses in *Atcerk1*. (a) Semi‐quantitative RT‐PCR analysis of *VvLYK1‐1*,* VvLYK1‐2* and *VvLYK1‐3* expression in leaf tissue of independently transformed *Atcerk1* lines. AtEF1α (At5g60390) was used as an internal control. (b) Activation of two mitogen‐activated protein kinases (MAPKs) 10 min after chitin treatment (1 mg/mL) detected by immunoblotting with an antibody raised against the human phosphorylated extracellular regulated protein kinase 1/2 (α‐pERK1/2). Homogeneous loading was checked by Ponceau red staining. Similar results were obtained in three independent experiments. (c) Relative expression of a defense gene encoding flagellin‐induced receptor kinase1 *(FRK1)* measured by qPCR, 2 h after chitin treatment (1 mg/mL). Data show a representative experiment from three independent biological ones. Means of the triplicate data were normalized by the housekeeping gene *At4g26410* and expressed as a percentage of the chitin‐treated WT Col‐0, set as 100%. Asterisks (*) indicate statistically significant differences between water and chitin treatment whereas hash marks (#) indicate statistically significant differences between WT or transgenic line and *Atcerk1*, using an unpaired heteroscedastic Student's *t* test (*P* < 0.05).

To investigate whether VvLYK1‐1, VvLYK1‐2 or VvLYK1‐3 are capable of activating chitooligosaccharide‐triggered defenses, expression constructs comprising each native *VvLYK1* coding sequence (i.e. no C‐terminal tag) under the control of a constitutive *35S* promoter were introduced into the *Atcerk1* mutant. Semi‐quantitative PCR was performed on the leaves of T2 transgenic lines to test for the presence of the *VvLYK1‐1*,* VvLYK1‐2* or *VvLYK1‐3* transcripts. Six transgenic lines were positively identified as expressing the *VvLYK1‐1* transgene and five transgenic lines were identified for *VvLYK1‐3* (Figure 3a). However, analysis of five independent transgenic lines, confirmed to contain the *VvLYK1‐2* construct by genomic PCR, indicated that transgene expression was either undetectable (lines #2, #12, #14) or at very low levels (lines #5 and #10) compared to *VvLYK1‐1* and *VvLYK1‐3* transgene expression (Figure 3a). The failure to positively identify lines highly expressing *VvLYK1‐2* suggested that this gene is potentially lethal when expressed under a strong constitutive *35S* promoter. This was confirmed by agro‐infiltration of the *p35S::VvLYK1‐2* construct into *N. benthamiana* leaves resulting in patchy necrosis after 48 h compared to leaf segments infiltrated with *Agrobacterium* alone (Figure S4). As *VvLYK1‐2* induces necrosis when over‐expressed, this suggests that it may have a crucial function in defense and its expression needs to be tightly regulated *in planta*. Based on these results, *Arabidopsis* lines transformed with the *p35S::VvLYK1‐2* construct were excluded from complementation analysis using a constitutive expression system.

Transgenic *Atcerk1/p35S::VvLYK1‐1* and *Atcerk1/p35S::VvLYK1‐3* lines were first examined for restoration of early chitin‐induced events by analysing the phosphorylation of MAPKs in two independent lines following treatment with chitin (NA‐COS‐Y; Lloyd *et al*., 2014) for 10 min prior to protein extraction. Figure 3b shows that chitin treatment triggered the phosphorylation of two MAPKs, with relative molecular weights of 43 and 47 kDa, in WT Col‐0 seedlings but no MAPK phosphorylation was observed in *Atcerk1*, in agreement with the previous report of Miya *et al*. (2007). Chitin‐induced MAPK activation was restored in the two independent *p35S::VvLYK1‐1* lines *#2* and *#5* (Figure 3b) but no MAPK phosphorylation was detected in protein samples extracted from the two independent *p35S::VvLYK1‐3* lines #1 and #2 (Figure 3b).

The expression of the defense gene encoding flagellin‐induced receptor kinase 1 (*FRK1)* was also investigated 2 h after chitin treatment. Chitin induced a high level of expression of *FRK1* in WT Col‐0 that was totally suppressed in the *Atcerk1* mutant (Figure 3c). *FRK1* expression was partly restored in the two *p35S::VvLYK1‐1* lines *#2* and *#5*, but remained close to the basal level in the two *p35S::VvLYK1‐3* lines *#1 and #2* (Figure 3c). Taken together, these results indicate that over‐expression of *VvLYK1‐1* can restore chitin‐triggered immune responses in *Atcerk1* but *VvLYK1‐3* cannot.

### *VvLYK1‐1* expression restores penetration resistance in *Atcerk1* against the non‐adapted powdery mildew *Erysiphe necator*

In addition to testing for complementation of MAPK activation and defense gene expression, the ability of *VvLYK1‐1* and *VvLYK1‐3* to restore resistance against a non‐adapted grapevine powdery mildew pathogen in the *Atcerk1* mutant was also determined. *Arabidopsis thaliana* is a non‐host for the fungus *E. necator*. Although a proportion of *E. necator* spores placed onto a Col‐0 leaf will successfully penetrate the epidermal cell wall and form a haustorium under the first appressorium, the pathogen is unable to complete its life cycle on this host (Feechan *et al*., 2013).

**Figure 4:**
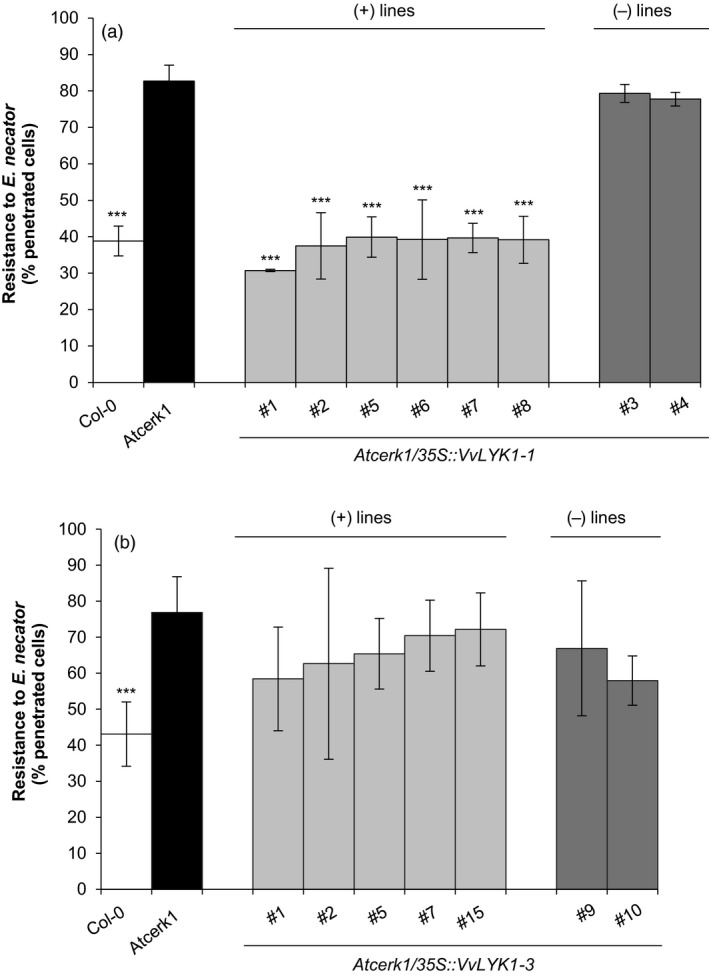
*VvLYK1‐1* expression restores penetration resistance against the non‐adapted powdery mildew *Erysiphe necator* in *Atcerk1*. Penetration efficiency (i.e. haustorium formation) of the non‐adapted powdery mildew pathogen *E. necator* on *Arabidopsis* WT (Col‐0), *Atcerk1* mutant and eight independent transgenic *Atcerk1* lines transformed with the *VvLYK1‐1* construct (a) or seven lines transformed with the *VvLYK1‐3* construct (b). One hundred germinated conidia were scored per leaf, with three leaves inoculated per line. Each data point represents the mean of three independent experiments ±SE. WT Col‐0 and transgenic lines were compared to the mutant *Atcerk1* with a Student *t*‐test (***, *P* < 0.001). (+) lines expressing the transgene. (−) lines with no detectable *VvLYK1* transcripts.

Figure 4a shows that the *Atcerk1* mutant is significantly more susceptible to penetration by *E. necator* than the WT Col‐0, showing the important role of AtCERK1 in non‐host resistance against non‐adapted powdery mildew species. More precisely, the penetration rate of WT Col‐0 leaves by *E. necator* spores ranged 35%–43% with a mean at 39% whereas in the *Atcerk1* mutant, the penetration rates ranged 76%–88% with a mean of 82% which approaches the rate of penetration by the adapted powdery mildew species *E*. *cichoracearum* on Col‐0 (Feechan *et al*., 2013). Constitutive expression of *VvLYK1‐1* in the *Atcerk1* mutant significantly reduced the mean penetration rates of *E. necator* in the leaves of all *Atcerk1/p35S::VvLYK1‐1* lines to levels comparable to the penetration rates on WT Col‐0 plants (31%–40%; Figure 4a). As additional negative controls, T2 lines #3 and #4 that had been generated through the same transformation procedure but had lost the introduced *VvLYK1‐1* transgene through segregation, showed mean penetration rates of *E. necator* similar to *Atcerk1* (77%–80%; Figure 4a). This demonstrates that the complementation of penetration resistance in the *Atcerk1/p35S::VvLYK1‐1* lines is a result of *VvLYK1‐1* expression and is not related to the transformation process. In contrast, expression of *VvLYK1‐3* in the five independent *Atcerk1/VvLYK1‐3* lines did not significantly reduce *E. necator* mean penetration rates (58%–72%; Figure 4b) in comparison to the *Atcerk1* mutant or the negative T2 control lines (#9 and #10; Figure 4b).

Together, the ability of VvLYK1‐1 to restore MAPK activation, the expression of *FRK1* and penetration resistance against *E. necator* in the *Atcerk1* mutant background suggests that VvLYK1‐1 mediates chitin sensing and might be important for grapevine defense against *E. necator*.

### The inducible expression of *VvLYK1‐2* also restores chitin‐triggered responses in the *Atcerk1* mutant

Due to the toxicity of constitutively expressed *VvLYK1‐2*, new constructs were generated in which *VvLYK1‐2* expression was driven by an inducible promoter. The pABindGFP vector (Bleckmann *et al*., 2010) permitted the inducible expression of a C‐terminally tagged VvLYK1‐2‐GFP fusion protein regulated by the β‐estradiol *LexA* promoter in the *Atcerk1* mutant background. Two independent hygromycin‐resistant T3 lines *Atcerk1/LexA::VvLYK1‐2‐GFP* #27 and #28 were selected to be homozygous and containing only one copy of the transgene.

**Figure 5:**
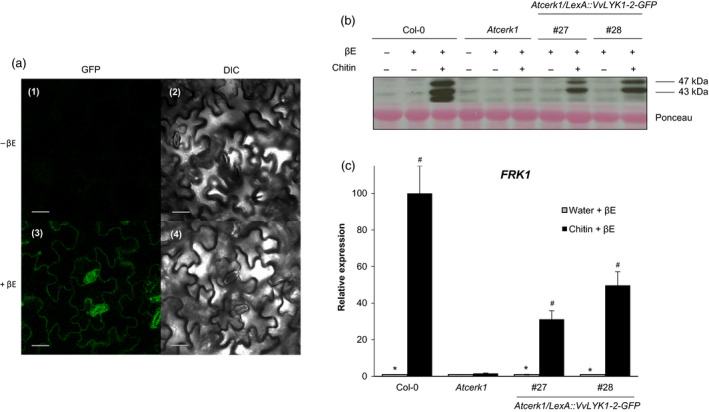
The inducible expression of VvLYK1‐2 also restores chitin‐triggered responses in *Atcerk1*. (a) Subcellular localization of *VvLYK1‐2‐GFP* visualized by confocal microscopy 4 h post‐treatment with β‐estradiol (βE). DIC, differential interference contrast. Bars, 20 μm. (b) Activation of two mitogen‐activated protein kinases (MAPKs) detected 10 min after chitin treatment (1 mg/mL) by immunoblotting with an antibody raised against the human phosphorylated extracellular regulated protein kinase 1/2 (α‐pERK1/2). Homogeneous loading was checked by Ponceau red staining. Similar results were obtained in three independent experiments. (c) Relative expression of the defense gene encoding flagellin‐induced receptor kinase1 *(FRK1)* measured by qPCR, 2 h post‐chitin treatment (1 mg/mL). Data show a representative experiment from three independent biological ones. Means of the triplicate data were normalized by the housekeeping gene *At4g26410* and expressed as a percentage of the transcript level in WT Col‐0 plants treated by chitin + β‐estradiol, set as 100%. Asterisks (*) indicate statistically significant differences between water and chitin treatment whereas hash marks (#) indicate statistically significant differences between WT or transgenic line and *Atcerk1*, using an unpaired heteroscedastic Student's *t* test (*P* < 0.05). For (b) and (c), inducible transgenic lines *Atcerk1/LexA::VvLYK1‐2‐GFP* were treated 1 h before chitin treatment with 10 μm β‐estradiol (βE).

Following β‐estradiol treatment, confocal microscopy confirmed the presence of the VvLYK1‐2‐GFP protein at the cell periphery (Figure 5a) suggesting a localization at the plasma membrane similar to VvLYK1‐1 (Figure 2d).

To investigate whether VvLYK1‐2 can also restore chitin‐induced signalling and immune responses in the *Atcerk1* mutant, MAPK activation and defense gene expression were analysed. Figure 5b shows that β‐estradiol pre‐treatment alone did not induce MAPK phosphorylation in the WT Col‐0 or in the *Atcerk1* mutant. However, β‐estradiol pre‐treatment followed by a chitin treatment lead to the restoration of MAPK phosphorylation in the two independent *Atcerk1*/*LexA::VvLYK1‐2‐GFP* lines #27 and #28 (Figure 5b). Similarly, the chitin‐induced expression of the defense gene *FRK1* was also restored in both lines *Atcerk1/pLexA::VvLYK1‐2‐GFP* #27 and #28 (Figure 5c). These data indicate that VvLYK1‐2, like VvLYK1‐1, also restores MAPK activation and immune gene expression in the *Atcerk1* mutant. Unfortunately, the use of this transient β‐estradiol‐inducible expression system did not permit us to obtain reproducible results concerning the putative role of VvLYK1‐2 in the resistance against *E. necator*.

### *VvLYK1‐1* and *VvLYK1‐2* expression restore chitosan‐triggered responses in the *Atcerk1* mutant

**Figure 6:**
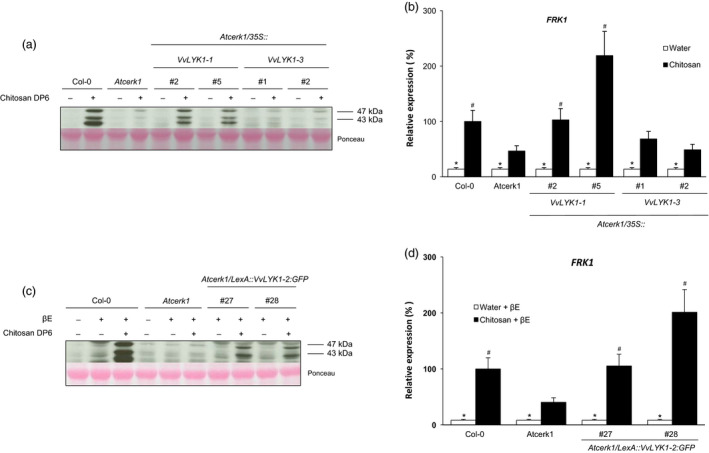
VvLYK1‐1 and VvLYK1‐2 expression restores chitosan‐triggered responses in the *Atcerk1* mutant. (a, c) Activation of two mitogen‐activated protein kinases (MAPKs) detected 10 min after treatment with chitosan DP6 (1 mg/mL) by immunoblotting with an antibody raised against the human phosphorylated extracellular regulated protein kinase 1/2 (α‐pERK1/2). Homogeneous loading was checked by Ponceau red staining. (b, d) Expression of a defense gene encoding the flagellin‐induced receptor‐like protein kinase 1 (*FRK1*) measured by qPCR 2 h after chitosan treatment. Data show an average of three biological experiments that were normalized by housekeeping gene *At4g26410* and compared with Col‐0 treated with chitosan, set as 100%. Asterisks (*) indicate statistically significant differences between water and chitosan treatment whereas hash marks (#) indicate statistically significant differences between WT or transgenic line and *Atcerk1*, using an unpaired heteroscedastic Student's *t* test (*P* < 0.05). (c, d) All lines were pretreated 1 h before elicitor treatment with β‐estradiol (βE; 10 μm), when indicated.

To further characterize these new grapevine PRRs, we also tested the responses triggered by chitosan in *Atcerk1/VvLYK1* transgenic lines (Figure 6). Like chitin (Figure 3), chitosan was able to strongly induce the phosphorylation of MAPKs in WT Col‐0 and this signalling pathway was highly compromised in the *Atcerk1* mutant (Figure 6a). Expression of *VvLYK1‐1* in the *Atcerk1* mutant also restored chitosan‐induced MAPK activation but *VvLYK1‐3* did not (Figure 6a). Similarly, the chitosan‐induced expression of the defense gene *FRK1* was also restored at the WT or higher level in both lines *Atcerk1/p35S::VvLYK1‐1* #2 and #5 whereas the *FRK1* transcript level in lines *Atcerk1/p35S::VvLYK1‐3* #1 and #2 was comparable to the one in *Atcerk1* (Figure 6b). Of note, the *FRK1* expression level in *Atcerk1* treated by chitosan is significantly higher than in the water control (Figure 6b). Chitosan‐induced phosphorylation of MAPKs (Figure 6c) and *FRK1* defense gene expression (Figure 6d) were also complemented in the two independent lines *Atcerk1/pLexA::VvLYK1‐2‐GFP* #27 and #28. Thus, VvLYK1‐1 and VvLYK1‐2 also restore chitosan‐triggered responses in *Atcerk1*.

## Discussion

Chitin is a well‐known PAMP which elicits typical immune responses in *Arabidopsis* (Cao *et al*., 2014; Miya *et al*., 2007; Petutschnig *et al*., 2010; Wan *et al*., 2008) and a number of other plant species (Akamatsu *et al*., 2013; Ao *et al*., 2014; Felix *et al*., 1998; Hayafune *et al*., 2014; Kaku *et al*., 2006; Liu *et al*., 2016; Shimizu *et al*., 2010; Zeng *et al*., 2012). However, little is known about chitin perception in grapevine. Here, we clearly demonstrate that chitin and chitosan, its deacetylated derivative, trigger grapevine immune responses such as phosphorylation of MAPKs and the expression of defense genes including *CHIT4C*,* STS*,* PAL* and *RBOHD*. Up‐regulation of genes encoding chitinases and PAL was also observed in *Arabidopsis* and rice upon chitin treatment (Kaku *et al*., 2006; Miya *et al*., 2007). Surprisingly, these chitooligosaccharides did not induce any detectable H_2_O_2_ production in grapevine, in contrast with *Arabidopsis* (Miya *et al*., 2007) and rice (Hayafune *et al*., 2014). However, this lack of H_2_O_2_ production clearly does not prevent the phosphorylation of MAPKs showing independence between these two pathways, in accordance with results previously obtained in *N. benthamiana* and *Arabidopsis* (Segonzac *et al*., 2011; Xu *et al*., 2014).

We also show that chitin enhances the resistance of grapevine leaves to the necrotrophic fungus *B. cinerea* and the obligate biotrophic oomycete *P. viticola* (Figure 1), as previously demonstrated following treatment with chitosan, flg22 or sulphated β‐1,3‐glucan (Aziz *et al*., 2006; Gauthier *et al*., 2014; Trdá *et al*., 2014). Similarly, treatment of rice plants with chitin reduced the susceptibility to the fungal pathogen *Magnaporthe oryzae* (Tanabe *et al*., 2006). More recently, chitin treatment was also shown to reduce the susceptibility of *Arabidopsis* to the bacterial pathogen *Pseudomonas syringae* pv tomato (*Pto*) DC3000 and the fungus *Alternaria brassicicola* (Cao *et al*., 2014). All of these results confirm that stimulation of plant immune responses with PAMPs can trigger enhanced resistance against different plant pathogens.

The degree of acetylation (DA) of chitooligosaccharides appeared to have no effect on the amplitude of the immune responses in grapevine although the duration of the chitosan‐triggered MAPK activation was longer (Figure 1). Similarly, in *Arabidopsis*, the DA of chitooligosaccharides had no effect on the activation of PAL (Cabrera *et al*., 2006). In contrast, in wheat, chitosan oligomers with a DA of 50% were better able to induce PAL activity than those possessing a DA of 0% (Vander *et al*., 1998). Thus, the structure/activity of chitooligomers might differ depending on the plant species used (Yin *et al*., 2016).

The activation of MAPKs and defense gene expression in grapevine cells treated with chitin demonstrates that grapevine possesses the cognate PRRs. Zhang *et al*. (2009) previously proposed the grapevine LYK family to be comprised of 12 members. We undertook a re‐examination of the predicted *LYK* gene family in grapevine in combination with published EST data and our own RNA‐Seq data. This revealed a number of errors in the original Zhang *et al*. (2009) predictions both in terms of the predicted ORFs and gene number. For example, the previously annotated single *VvLYK10* gene (Zhang *et al*., 2009) was found to contain a tandemly arrayed *LYK* gene pair. The two ORFs encode proteins with a 74% amino acid similarity to each other and homology to AtLYK5 (49% and 51% amino acid similarity). Tandem *LYK* gene pairs have also been identified in legume and poplar plants (Zhang *et al*., 2009). We are therefore proposing a new annotation scheme for the grapevine *VvLYK* gene family which uses a naming convention based on sequence similarity to *Arabidopsis LYK* gene family (Figure 2a, Table S1).

In *Arabidopsis*, AtCERK1/LYK1 has been demonstrated to play a key role in chitin‐induced signalling. Grapevine encodes three putative orthologs of AtCERK1/LYK1, designated VvLYK1‐1, VvLYK1‐2 and VvLYK1‐3. Our data demonstrate that the constitutive expression of *VvLYK1‐1* or the inducible expression of *VvLYK1‐2* in the *Atcerk1* mutant restores chitooligosaccharide‐induced immune responses such as MAPK activation and expression of the defense gene *FRK1*. Thus our results demonstrate that these two independent grapevine proteins are functional orthologs of AtCERK1/LYK1, suggesting duplication events during the evolution of the ancestral genome of *V. vinifera* (Jaillon *et al*., 2007). VvLYK1‐1 and/or VvLYK1‐2 also restore chitosan perception by the *Atcerk1* mutant, as indicated by MAPK activation, suggesting that in grapevine the same PRRs can mediate both chitin and chitosan signalling. Similarly, an AtCERK1 protein band shift was detected in *Arabidopsis* after treatment with chitin or chitosan and the ectodomain of AtCERK1 has been shown to bind chitosan DP6 (Petutschnig *et al*., 2010). However, the fact that ROS production in *Arabidopsis* is induced by chitin DP6 but not by the fully deacetylated chitosan DP6 (Figure S1) confirmed previous results of Gubaeva *et al*. (2018) indicating that some downstream signalling events may be divergent. When the fact that a weak signal for MAPKs activation and a significant *FRK1* transcript accumulation are observed in the *Atcerk1* mutant following chitosan treatment (Figure 6) is considered together with previous results demonstrating AtCERK1/LYK1‐independent defense gene expression (Povero *et al*., 2011), it suggests that different AtLYK proteins may be involved in detecting different chitooligosaccharides. A preliminary investigation of the response of five different *Atlyk* mutants to chitosan DP6 treatment shows that MAPK activation is weaker in the *Atlyk5* and *Atlyk3* mutants compared to WT Col‐0 (Figure S5) suggesting that the AtLYK3 and AtLYK5 proteins might also participate in the perception of chitosan oligomers in combination with AtCERK1/LYK1.

Interestingly, we were unable to obtain *Arabidopsis* lines with high levels of constitutively expressed *VvLYK1‐2*. Furthermore, we observed an induction of cell death following transient expression of *VvLYK1‐2* in tobacco (Figure S4), confirming gene toxicity. Cell death in response to heterologous LysM‐RK expression in *N. benthamiana* has previously been observed when *AtCERK1* was fused with the yellow fluorescent protein variant, sYFP2 and transiently expressed under the control of a *35S* promoter (Pietraszewska‐Bogiel *et al*., 2013), demonstrating the importance of regulating *LYK* expression levels.

In order to confirm the results obtained from complementation studies in *Arabidopsis*, we also attempted to confirm the function of *VvLYK1‐1* and *VvLYK1‐2* in chitin and chitosan perception in grapevine by generating grapevine transgenics in which these genes had been silenced. However, no transformed calli were recovered in three independent agrobacterium‐mediated transformations of somatic grapevine embryos with *p35S::antisense‐VvLYK1‐1* and *p35S::antisense‐VvLYK1‐2* constructs whereas parallel control transformations with a *p35S::GFP construct* were successful (data not shown).

*VvLYK1‐1* expression in the *Atcerk1* mutant background was demonstrated to restore non‐host resistance against grapevine powdery mildew suggesting that VvLYK1‐1 may participate in anti‐fungal basal resistance also in grapevine. As the *Atcerk1* mutant is more susceptible to the non‐adapted pathogen *E. necator*, it also indicates that AtCERK1/LYK1 plays a role in the non‐host resistance against this grapevine pathogen. Paparella *et al*. (2014) previously showed that an *Atlyk3‐1* mutant was more resistant to *B. cinerea* suggesting that AtLYK3 negatively regulates certain immune responses such as the production of phytoalexins, suggesting that different members of the AtLYK gene family may play a role in the basal resistance of *Arabidopsis* against different fungal pathogens. It is interesting to note that *VvLYK4‐1/2*,* VvLYK5‐1* and *VvLYK6* genes are highly up‐regulated during *B. cinerea* infection of grapevine berries (Figure 2). Thus, it is plausible that other members of the large VvLYK family may exhibit specificity to the different ligands released during the interactions of grapevine with this kind of pathogen.

Cao *et al*. (2014) recently showed that AtLYK5 is able to bind chitin at a greater affinity than AtCERK1 and that chitin perception leads to the formation of an AtCERK1‐AtLYK5 dimer which is required for AtCERK1 phosphorylation. These observations led them to propose that AtLYK5, and not AtCERK1, is the primary receptor for chitin perception and which has been proposed to be responsible for the activation of defense responses (Cao *et al*., 2014). Thus, one explanation of our complementation data is that VvLYK1‐1 or VvLYK1‐2 could dimerize with AtLYK5 in the presence of chitin, but that the interaction is not sufficiently effective to obtain a full restoration of MAPK activation and defense gene expression back to wild‐type levels. This suggests the existence of molecular complexes for chitooligosaccharides perception in grapevine, as previously shown for rice (Hayafune *et al*., 2014) and *Arabidopsis* (Cao *et al*., 2014).

In summary, we present a re‐annotation of the *VvLYK* gene family and demonstrate that two *AtCERK1/LYK1* orthologs, *VvLYK1‐1* and *VvLYK1‐2,* are involved in chitooligosaccharide signalling. Elucidating components of PAMP‐triggered immunity in grapevine opens the possibility of developing grapevine varieties with durable resistance against fungal pathogens. *E. necator* has adapted to successfully infect grapevine by evolving host‐specific effector proteins that target and re‐programme the signalling pathways that lead to PAMP‐triggered immunity. The introduction of PRRs from a closely related species that can function in *V. vinifera* but are not modulated by *E. necator*'s specific effector suite has the potential to restore PAMP‐triggered immunity against this adapted pathogen (Heath, 2000; Lee *et al*., 2016). The proof of concept for this approach was demonstrated by the expression of the *Arabidopsis* PRR EFR in *N. benthamiana*, tomato, rice and wheat plants which conferred greater resistance against a range of phytopathogenic bacteria (Lacombe *et al*., 2010; Lu *et al*., 2015; Schoonbeek *et al*., 2015; Zipfel *et al*., 2006). Several components of the chitin‐signalling network are known targets of numerous pathogen effector proteins (van den Burg *et al*., 2006; van Esse *et al*., 2007, 2008; Gimenez‐Ibanez *et al*., 2009; Mentlak *et al*., 2012; Yamaguchi *et al*., 2013; Zeng *et al*., 2012), supporting the hypothesis that the chitin‐signalling network is an excellent candidate for enhancing the grapevine immune response.

Thus, further experiments will be necessary to gain a better understanding of how grapevine cells specifically perceive different chitooligosaccharides *via* these complex receptors and to determine the role of each member of the *VvLYK* multigene family, particularly during its interactions with both beneficial and pathogenic microbes.

## Experimental procedures

### Plant, cell culture and fungal materials

*Arabidopsis thaliana* wild‐type (WT) Columbia (Col‐0), mutant *Atcerk1* (GABI‐Kat_096F09, allele *Atcerk1‐2*; (Gimenez‐Ibanez *et al*., 2009) or transgenic lines *Atcerk1/35S::VvLYK1‐1/3* and *Atcerk1/LexA::VvLYK1‐2‐GFP* were grown under a 10/14‐h day/night cycle at 20/18 °C (Trdá *et al*., 2014). For *in vitro* culture, *Arabidopsis* plants were grown on solid or in liquid half Murashige and Skoog (MS) medium including Nitsch vitamins (M0256; Duchefa, Haarlem, the Netherlands) supplied with 10 g/L sucrose. Seedlings were grown at 20 °C (day) or 18 °C (night) with a 14‐h photoperiod.

Grapevine (*V. vinifera* cvs Cabernet Sauvignon and Marselan) cuttings were grown in a greenhouse until they had developed 6–8 leaves. The second and third youngest adult leaves from each plant were used for experiments, as previously indicated (Steimetz *et al*., 2012). Grapevine cells (*V. vinifera* cv. Gamay) were cultivated as described in Vandelle *et al*. (2006). For all experiments, 7‐day‐old cultures were diluted twice with new medium 24 h prior to use.

Grapevine powdery mildew (*E. necator –* isolate APC) was maintained detached leaves of *V. vinifera* cv. Cabernet Sauvignon as previously described (Donald *et al*., 2002). Grapevine downy mildew (*P. viticola* – isolate collected from a Burgundy vineyard) was routinely maintained on *V. vinifera* cv. Marselan plants as previously described (Steimetz *et al*., 2012).

### Elicitors

Chitin and chitosan hexamer, with a degree of acetylation (DA) of 99.9% and 0.1%, respectively, were provided by Elicityl (Crolles, France). They were extracted from exoskeletons of crustaceans, hydrolysed, purified by chromatography and finally their degree of polymerization (DP) and DA were verified by ^1^H NMR analysis. The crab shell chitin NA‐COS‐Y (Lloyd *et al*., 2014), was obtained from Yaizu Suisankagaku Industry Co. (Yaizu, Japan). All the above mentioned chitooligosaccharides were dissolved in sterile ultrapure water (pH 8.5) at a concentration of 1 or 10 mg/mL. Sulphated laminarin (PS3), used as a potent inducer of grapevine resistance (Gauthier *et al*., 2014), was provided by Goëmar Laboratories and dissolved in sterile ultrapure water.

The flagellin‐derived flg22 peptide from *Xanthomonas campestris* pv *campestris* strain 305 (QRLSSGLRINSAKDDAAGLAIS) was purchased from Proteogenix and dissolved in sterile ultra‐pure water at 1 mm, as previously described (Trdá *et al*., 2014).

### MAPK activation

Grapevine cells were equilibrated as described in Dubreuil‐Maurizi *et al*. (2010), then treated with chitooligosaccharides (100 μg/mL) or water (as control) and harvested at 0, 5, 10, 20, 40 and 60 min post‐treatment. MAPK activation was detected after immunoblotting of the extracted proteins using anti‐p42/44‐phospho‐ERK antibody (Cell Signaling, Danvers, MA). Transfer quality and homogeneous loading were checked by Ponceau red staining.

For *Arabidopsis* plantlets, 10‐ to 15‐day‐old liquid‐grown seedlings were equilibrated for 24 h in fresh half MS medium. β‐estradiol (10 μm) was added 1 h before elicitor treatment (1 mg/mL) for inducible transgenic lines *Atcerk1/LexA::VvLYK1‐2‐GFP*. Seedling samples were harvested 10 min after chitin or chitosan treatment.

### Analysis of defense gene expression by quantitative polymerase chain reaction (qPCR)

For defense gene expression kinetics using grapevine cell suspensions, the cell culture density was adjusted to 0.1 g FWC/mL with NN medium, 16 h prior to experiment. Cells were then treated with 100 μg/mL chitooligosaccharides or water (as control) and harvested at 1 h post‐treatment by filtration on GF/A filters.

For *Arabidopsis*, 10‐ to 15‐day‐old seedlings grown on solid half MS medium were transferred in liquid medium 2 days before treatment in a 24‐well microtitre plate. β‐estradiol (10 μm) was added 1 h before treatment with 1 mg/mL of chitooligosaccharides for 2 h.

For both cells and seedlings, tissues were briefly ground before the addition of TRIzol^®^ (Invitrogen, Life Technologies, Saint‐Aubin, France). RNA extraction was then carried out following the manufacturer's instructions (Invitrogen). Reverse transcription was performed using Superscript III (Invitrogen) for cells or M‐MLV reverse transcriptase (Invitrogen) for seedlings, following the manufacturer's protocol. Real‐time qPCR was carried out as described previously (Trdá *et al*., 2014), except that a 1:100 dilution of cDNA was used. The relative transcript level was calculated using the comparative ΔΔCt method (Livak and Schmittgen, 2001) with the previously validated grapevine *VvEF1*α (Dubreuil‐Maurizi *et al*., 2010; Reid *et al*., 2006) or the *Arabidopsis At4g26410* (Czechowski *et al*., 2005) housekeeping gene as internal control for normalization (*AtOLI* in Table S3).

### Confocal microscopy

Confocal microscopy was performed using a Leica TCS SP2‐AOBS confocal laser scanning microscope with a 40X oil‐immersion objective (numerical aperture 1.25; Leica, Nanterre, France). Inducible transgenic lines were sprayed with 200 μm β‐estradiol, 4 h before visualization. Leaf segments were mounted in ultra‐pure water or in 1 m NaCl solution for plasmolysis experiments. For FM4‐64 staining, samples were incubated in 8 μm FM4‐64 solution in water for 10 min prior to observation. Fluorescent markers were visualized at 488 nm. GFP and FM4‐64 emissions were bandpass filtered at 500–525 nm and 616–694 nm, respectively.

### Botrytis and downy mildew assays

Leaves from the second and third adult top leaves of at least three grapevine plants were first sprayed on both sides with elicitor solution in 0.1% surfactant (Deshcofix) or surfactant alone (control) for 48 h.

For *B. cinerea* infection assays, 36 leaf discs (1.9 cm diameter) were incubated on moist Whatmann paper and inoculated on the upper surface with 1000 conidia in a 20 μL‐droplet of potato dextrose broth (PDB), ¼ diluted. Inoculated discs were placed in a plastic box maintained in 100% humidity under a 10/14 h day/night cycle at 20/18 °C. Infection intensity was assessed 3 days post‐inoculation (dpi) by measuring the macerated lesion diameter.

For *P. viticola* infection, the lower leaf surface was sprayed with a freshly prepared suspension (2.10^4^ sporangia/mL) and plants were maintained in 100% humidity for 2 h. Leaf discs (1 cm diameter) were cut, transferred onto moist Whatmann paper in a plastic box and maintained in 100% humidity under a 10/14 h day/night cycle at 20/18 °C. Infection intensity was assessed at 8 dpi by measuring the sporulating area using image analysis Visilog 6.9 software (Kim Khiook *et al*., 2013).

### Powdery mildew penetration assay on transgenic *Arabidopsis*

Four‐week‐old *Arabidopsis* plants were used to assess powdery mildew penetration efficiency. Two leaves per plant were infected with *E. necator* using a fine paintbrush. Detached leaf material was sampled 48 hpi and stained with trypan blue according to Koch and Slusarenko (1990). Fungal structures were visualized using a Zeiss (Göttingen, Germany) Axioscop 2 light microscope. A minimum of 100 germinated spores were scored on each leaf. Successful penetration of epidermal cells (% penetrated cells) was indicated by the presence of a haustorium or a secondary hyphae.

### Phylogenetic analysis of the VvLYK family

Proteins were aligned with the CLUSTAL W program (Tables S1 and S2). The Maximum Likelihood phylogenetic tree was generated with the MEGA7 software (Kumar *et al*., 2016), using a bootstrapping of 1000 replications.

### Expression analysis of *VvLYK* genes in pathogen‐infected grape tissues

Young glossy *V. vinifera* cv. Cabernet Sauvignon leaves of similar developmental stage (~6 cm in diameter) were inoculated with *E. necator* conidia as described previously (Donald *et al*., 2002). The leaves were incubated at 23 °C under a 16 h light/8 h dark cycle and sampled at 0, 6, 12 and 24 hpi into liquid nitrogen. Total RNA was extracted from two independent leaves at each time point using the Spectrum Plant Total RNA Kit (Sigma‐Aldrich, St. Louis, MO) and DNase‐treated according to the manufacturer's instructions. RNA quantity and quality were assessed using a 2100 Bioanalyzer (Agilent Technologies, Waldbronn, Germany). Library construction and Illumina RNA sequencing (single end, 100 bp reads) were carried out at the Australian Genome Research Facility (Melbourne, Australia). Reads were mapped to the coding sequences of each predicted *VvLYK* cDNA sequence using CLC Genomics Workbench v6.0.1. Reads were normalized according to (i) length of the VvLYK reference sequence and (ii) mean relative expression of *V. vinifera* cv Cabernet sauvignon housekeeping genes: elongation factor 1‐alpha (XM_002284888); glyceraldehyde‐3‐phosphate dehydrogenase (XM_002263109), phosphoenolpyruvate carboxylase (XM_010658735), to produce a Relative Expression Value (REV) at each time point of infection.

For microarray data, RNA was extracted from grape berries infected with *B. cinerea* then microarray hybridization and data analysis were performed as described in Kelloniemi *et al*. (2015). All microarray expression data are available at GEO under the entry GSE65969.

### Generation of the *Atcerk1/VvLYK1* transgenic lines

The coding sequences of *VvLYK1‐1*,* VvLYK1‐2* and *VvLYK1‐3* from *V. vinifera* cv. Cabernet Sauvignon were amplified from grapevine leaf cDNA prepared as previously described (Feechan *et al*., 2013). Gene‐specific primers were designed with 5′‐*Xho* I or *Xba* I restriction sites to facilitate subcloning (Table S3). Amplified products of the expected size were cloned into pCR‐BLUNT vector and verified by sequencing. The coding sequences were subcloned into pART7 vector (Gleave, 1992) between the *35S* promoter and *OCS* terminator sequences. The *35S‐VvLYK1‐OCS* expression cassettes were subcloned as *Not* I fragments into the binary vector pART27 and then transferred into *Agrobacterium tumefaciens* strain EHA‐105 for *Arabidopsis* transformation or *A. tumefaciens* strain GV3101 for agroinfiltration experiments (Williams *et al*., 2016).

The GFP‐tagged constructs were amplified using primers designed to replace the stop codon with an Ala codon (GCC nucleotides, Table S3). PCR products of the expected size were first directionally subcloned into pENTR™/D‐TOPO^®^ vector (Invitrogen), then inserted into Gateway expression vectors (Karimi *et al*., 2002) by using Gateway LR Clonase™ II enzyme mix (Invitrogen). The three full‐length coding sequences of *VvLYK1‐1*,* VvLYK1‐2* and *VvLYK1‐3* were cloned into pK7FWG2 (kanamycin resistance) to obtain a constitutive overexpression construct (*p35S::VvLYK1‐1/‐2/‐3‐GFP*) or in pABindGFP (Bleckmann *et al*., 2010; hygromycin resistance) for a β‐estradiol inducible gene expression (*pLexA::VvLYK1‐2‐GFP*).

The *Arabidopsis Atcerk1* mutant (Gimenez‐Ibanez *et al*., 2009) was transformed using the floral dip method (Clough and Bent, 1998). Antibiotic resistant transgenic plants were screened in the T1 generation as described previously (Zipfel *et al*., 2006).

For analysis of *VvLYK1‐1/‐2/‐3* transgene expression in the T2 generation, seed collected from selfed T1 lines was sown into soil and plants grown in a controlled growth chamber under a 10/14 h day/night cycle at 24 °C. Leaf material (~50 mg) was sampled from individual T2 segregating lines and the presence of the *VvLYK1‐1/‐2/‐3* transgene confirmed by genomic PCR. Positive lines were resampled for total RNA extraction, cDNA synthesis and semi‐quantitative PCR analysis of *VvLYK1‐1*,* VvLYK1‐2* or *VvLYK1‐3* transcript expression using primers listed in Table S3.

## Accession numbers

*Vitis vinifera* cv. Cabernet Sauvignon sequences: *VvLYK1‐1* (MF177032), *VvLYK1‐2* (MF177033), *VvLYK1‐3* (MF177034). *VvLYK* sequences fused with Cter‐GFP tag: *VvLYK1‐1‐GFP* (MF537036), *VvLYK1‐2‐GFP* (MF537037) and *VvLYK1‐3‐GFP* (MF537038).

## Funding

This work has been financially supported by ANR (PATRIC project, grant ANR‐13‐KBBE‐0001) (to BP), the Regional Council of Bourgogne Franche‐Comté (PARI grant 2016‐9201AAO050S01636 and FEDER grant BG0005888) and INRA for the funding of Justine Claverie's PhD (grants 2015‐9201AAO048502578 and 29000907), by the Gatsby Charitable Foundation (to CZ), and by the Biotechnology and Biological Sciences Research Council (BBSRC) grants BB/G024936/1 (ERA‐PG PRR CROP (to CZ).

## Author contributions

DB, CV, LJD performed most of the experiments; LT, FB, CZ and BP conceived the original screening and research plans; JC, AC, BD and LS provided technical assistance; MCH, FB, CZ and BP supervised the experiments, LT, DB, LJD and PT designed the experiments and analysed the data; DB, LJD, IBD and BP conceived the project and wrote the article with contributions of all the authors; FB, MA and CZ supervised and complemented the writing.

## Conflict of interest

The authors declare no conflict of interest.

## Supporting information


**Figure S1** Chitin and chitosan DP6 did not induce similarly ROS production in *Arabidopsis* and grapevine cells.
**Figure S2** The crab shell chitin also induced defense responses in grapevine cells.
**Figure S3** Alignment of AtCERK1/LYK1, the rice OsCERK1 and its putative orthologs in grapevine (VvLYK1‐1/‐2/‐3).
**Figure S4** Necrosis observed in response to the over‐expression of *VvLYK1‐2* in *Nicotiana benthamiana*.
**Figure S5** Immunodetection of MAPKs in *Arabidopsis* mutants *Atlyk1‐5* in response to chitosan.
**Table S1** The *Vitis vinifera* VvLYK family contains 15 putative genes in the grapevine genome.
**Table S2** Percentage of amino acid identity or similarity between VvLYK1‐1/‐2/‐3 and AtCERK1/LYK1 or OsCERK1.
**Table S3** Primers used in this study.
