# Supplementary material for: The grapevine (Vitis vinifera) LysM receptor kinases VvLYK1‐1 and VvLYK1‐2 mediate chitooligosaccharide‐triggered immunity
Source: Plant Biotechnol J. 2018 Oct 22;17(4):812–25. doi: 10.1111/pbi.13017 (PMC6419575; doi:10.1111/pbi.13017)
Supplement: Supplementary file 1 — Figure S1 Chitin and chitosan DP6 did not induce similarly ROS production in Arabidopsis and grapevine cells. Figure S2 The crab shell chitin also induced defense responses in grapevine cells. Figure S3 Alignment of AtCERK1/LYK1, the rice OsCERK1 and its putative orthologs in grapevine (VvLYK1‐1/‐2/‐3). Figure S4 Necrosis observed in response to the over‐expression of VvLYK1‐2 in Nicotiana benthamiana. Figure S5 Immunodetection of MAPKs in Arabidopsis mutants Atlyk1‐5 in response to chitosan. Table S1 The Vitis vinifera VvLYK family contains 15 putative genes in the grapevine genome. Table S2 Percentage of amino acid identity or similarity between VvLYK1‐1/‐2/‐3 and AtCERK1/LYK1 or OsCERK1. Table S3 Primers used in this study. [file PBI-17-812-s001.pdf]

Supplemental Figures

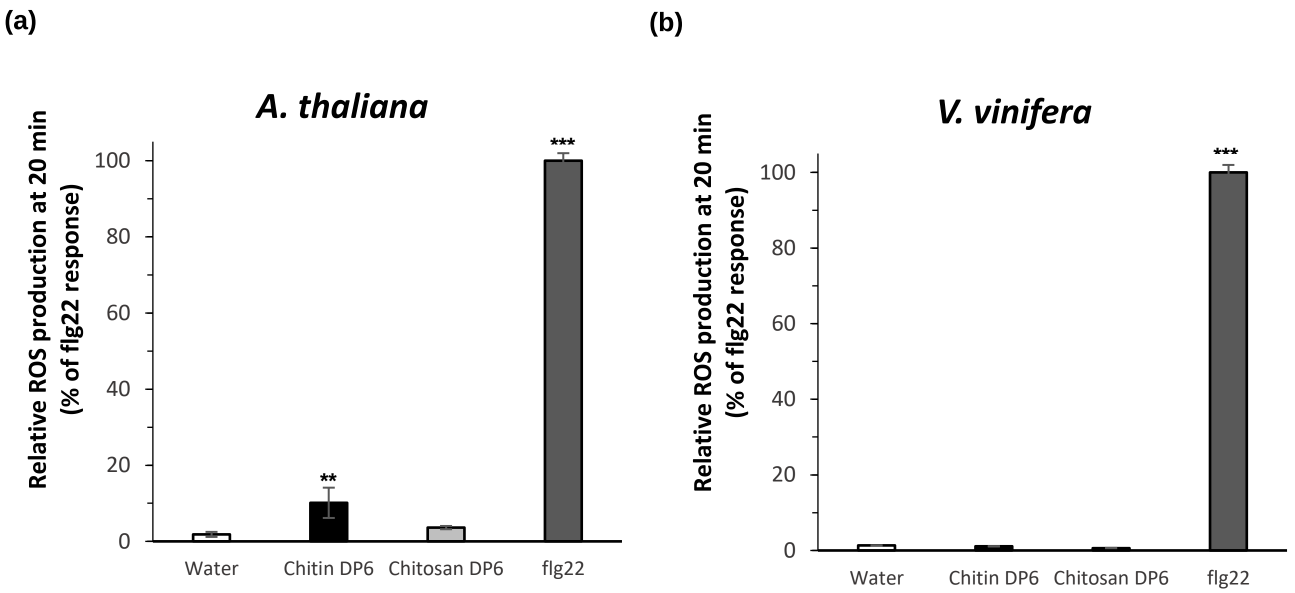

Fig. S1. Chitin and chitosan DP6 did not induce similarly ROS production in *Arabidopsis* and grapevine cells. Relative H_2O_2 production in **(a)** *A. thaliana* or **(b)** *V. vinifera* cells 20 min post-treatment with chitin DP6 (100 µg mL^{-1}), chitosan DP6 (100 µg mL^{-1}) and flg22 (1 µM) treatments or water (negative control). ROS production was measured by chemiluminescence of luminol according to Trdá et al. (2014) and results are expressed relatively to flg22 (positive control=100%). Values are means ± SE from three independent experiments (n=3). Asterisks indicate a statistically significant difference between control and the elicitor treatment (Tukey's pairwise test; *, $P < 0.05$, **, $P < 0.01$, ***, $P < 0.001$).

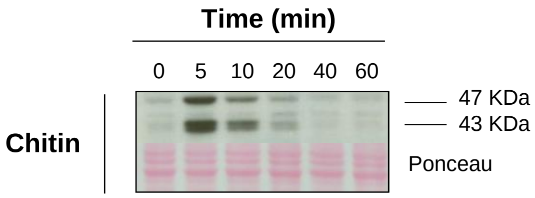

Fig. S2. The crab shell chitin also induced defense responses in grapevine cells. Activation kinetics of two mitogen-activated protein kinases (MAPKs) in grapevine cells treated with 100 µg.mL^{-1} of the unpurified crab shell chitin NA-COS-Y (Lloyd et al., 2014). MAPKs were detected by immunoblot with an antibody raised against the human phosphorylated extracellular regulated protein kinase 1/2 (α-pERK1/2). Fifteen µg of proteins were loaded in each lane. Homogeneous loading was checked by Ponceau red staining. This result shows one representative experiment out of three.

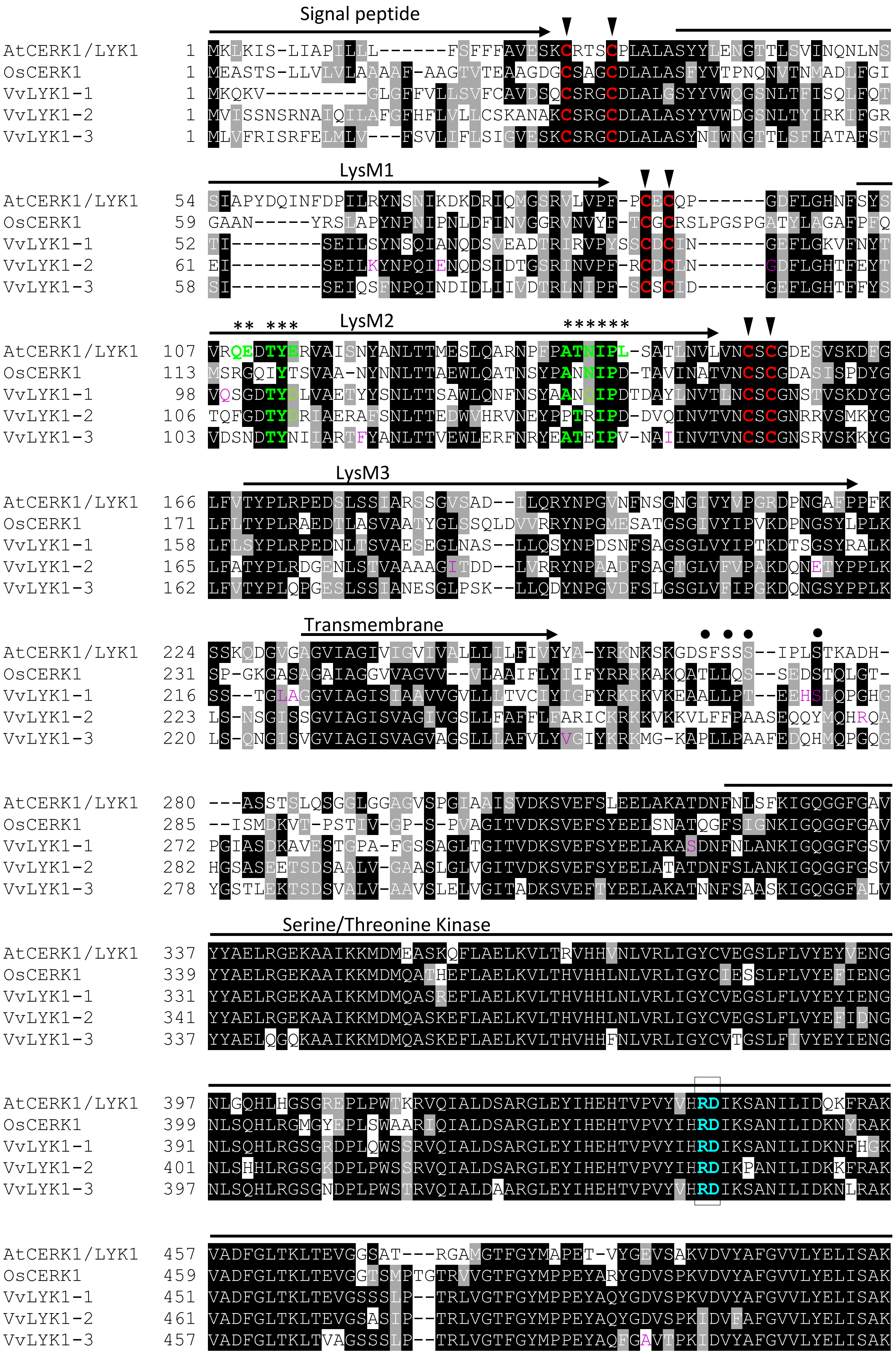

Signal peptide
AtCERK1/LYK1 1 MKLKIS-LIAPILLL------FSFFFAVESKCRTSCPLALASYYLENGTTLSVINQNLNS
OsCERK1 1 MEASTS-LLVLVLAAAAF-AAGTVTEAAGDGCSAGCDLALASFYVTPNQNVTNMADLFGI
VvLYK1-1 1 MKQKV----------GLGFFVLLSVFCAVDSQCSRGCDLALGSYYVWQGSNLTFISQLFQT
VvLYK1-2 1 MVISSNSRNAIQILAFGFHFLVLLCSKANAKCSRGCDLALASYYVWDGSNLTYIRKIFGR
VvLYK1-3 1 MLVFRISRFELMLV---FSVLIFLSIGVESKCSRGCDLALASYNIWNGTTLSFIATAFST
LysM1
AtCERK1/LYK1 54 SIAPYDQINFDPILRYNSNIKDKDRIQMGSRVLVPF-PCECQP------GDFLGHNFSYS
OsCERK1 59 GAAN-----YRSLAPYNPNIPNLDFINVGGRVNVYF-TCGCRSLPGSPGATYLAGAFPFQ
VvLYK1-1 52 TI---------SEILSYNSQIANQDSVEADTRIRVPYSSCDCIN------GEFLGKVFNYT
VvLYK1-2 61 EI---------SEILKYNPQIENQDSIDTGSRINVPF-RCDCLN------GDFLGHTFEYT
VvLYK1-3 58 SI---------SEIQSFNPQINDIDLIIVDTRLNIPF-SCSCID------GEFLGHTFFYS
** *** LysM2 ******
AtCERK1/LYK1 107 VRQEDTYERVAISNYANLTTMESLQARNPFPATNIPL-SATLNVLVNCSCGDESVSKDFG
OsCERK1 113 MSRGQIYTSVAA-NYNNLTTAEWLQATNSYPANNIPD-TAVINATVNCSCGDASISPDYG
VvLYK1-1 98 VQSGDTYDLVAETYYSNLTTSAWLQNFNSYAANQIPDTDAYLNVTLNCSCGNSTVSKDYG
VvLYK1-2 106 TQFGDTYDRIAERAFSNLTTEDWVHRVNEYPPTRIPD-DVQINVTVNCSCGNRRVSMKYG
VvLYK1-3 103 VDSNDTYNIIARTFYANLTTVEWLERFNRYEATEIPV-NAIINVTVNCSCGNSRVSKKYG
LysM3
AtCERK1/LYK1 166 LFVTYPLRPEDSLSSIARSSGVSAD--ILQRYNPGVNFNSGNGIVYVPGRDPNGAFPPFK
OsCERK1 171 LFLTYPLRAEDTLASVAATYGLSSQLDVVRRYNPGMESATGSGIVYIPVKDPNGSYLPLK
VvLYK1-1 158 LFLSYPLRPEDNLTSVAESEGLNAS--LLQSYNPDSNFSAGSGLVYIPTKDTSGSYRALK
VvLYK1-2 165 LFATYPLRDGENLSTVAAAAGITDD--LVRRYNPAADFSAGTGLVFVPAKDQNETYPPLK
VvLYK1-3 162 LFVTYPLQPGESLSSIANESGLPSK--LLQDYNPGVDFSLGSGLVFIPGKDQNGSYPPLK
Transmembrane
AtCERK1/LYK1 224 SSKQDGVGAGVIAGIVIGVIVALLLILFIVYYA-YRKNKSKGDSFSSS--IPLSTKADH-
OsCERK1 231 SP-GKGASAGAIAGGVVAGVV--VLAAIFLYIIFYRRRKAKQATLLQS--SEDSTQLGT-
VvLYK1-1 216 SS--TGLAGGVIAGISIAAVVGVLLLTVCIYIGFYRKRKVKEAALLPT--EEHSLQPGHG
VvLYK1-2 223 LS-NSGISSGVIAGISVAGIVGSLLFAFFLFARICKRKKVKKVLFFPAASEQQYMQHRQA
VvLYK1-3 220 LS-QNGISVGVIAGISVAGVAGSLLIAFVLYVGIYKRKMG-KAPLLPAAFEDQHMQPGQG
AtCERK1/LYK1 280 ---ASSTSLQSGGLGGAGVSPGIAAISVDKSVEFSLEELAKATDNFNLSFKIGQGGFGAV
OsCERK1 285 --ISMDKVT-PSTIV-GP-S-PVAGITVDKSVEFSYEELSNATQGFSIGNKIGQGGFGAV
VvLYK1-1 272 PGIASDKAVESTGPA-FGSSAGLTGITVDKSVEFSYEELAKASDNFNLANKIGQGGFGSV
VvLYK1-2 282 HGSASEETSDSAALV-GAASLGLVGITVDKSVEFSYEELATATDNFSLANKIGQGGFGSV
VvLYK1-3 278 YGSTLEKTSDSVALV-AAVSLELVGITADKSVEFTYEELAKATNNFSAASKIGQGGFALV
Serine/Threonine Kinase
AtCERK1/LYK1 337 YYAELRGEKAAIKKMDMEASKQFLAELKVLTRVHHVNLVRLIGYCVEGSLFLVYEYVENG
OsCERK1 339 YYAELRGEKAAIKKMDMQATHEFLAELKVLTHVHHLNLVRLIGYCIESSLFLVYEFIENG
VvLYK1-1 331 YYAELRGEKAAIKKMDMQASREFLAELKVLTHVHHLNLVRLIGYCVEGSLFLVYEYIENG
VvLYK1-2 341 YYAELRGEKAAIKKMDMQASKEFLAELKVLTHVHHLNLVRLIGYCVEGSLFLVYEFIDNG
VvLYK1-3 337 YYAELQGQKAAIKKMDMQASKEFLAELKVLTHVHHFNLVRLIGYCVTGSLFIVYEYIENG
AtCERK1/LYK1 397 NLGQHLHGSGREPLPWTKRVQIALDSARGLEYIHEHTVPVYVHRDIKSANILIDQKFRAK
OsCERK1 399 NLSQHLRGMGYEPLSWAARIQIALDSARGLEYIHEHTVPVYIHRDIKSANILIDKNYRAK
VvLYK1-1 391 NLSQHLRGSGRDPLQWSSRVQIALDSARGLEYIHEHTVPVYIHRDIKSANILIDKNFHGK
VvLYK1-2 401 NLSHHLRGSGKDPLPWSSRVQIALDSARGLEYIHEHTVPVYIHRDIKPANILIDKKFRAK
VvLYK1-3 397 NLSQHLRGSGNDPLPWSTRVQIALDAARGLEYIHEHTVPVYVHRDIKSANILIDKNLRAK
AtCERK1/LYK1 457 VADFGLTKLTEVGGSAT---RGAMGTFGYMAPET-VYGEVSAKVDVYAFGVVLYELISAK
OsCERK1 459 VADFGLTKLTEVGGTSMPTGTRVVGTFGYMPPEYARYGDVSPKVDVYAFGVVLYELISAK
VvLYK1-1 451 VADFGLTKLTEVGSSSLP--TRLVGTFGYMPPEYAQYGDVSPKVDVYAFGVVLYELISAK
VvLYK1-2 461 VADFGLTKLTEVGSASIP--TRLVGTFGYMPPEYAQYGDVSPKIDVFAFGVVLYELISAK
VvLYK1-3 457 VADFGLTKLTVAGSSSLP--TRLVGTFGYMPPEYAQFGAVTPKIDVYAFGVVLYELISAK

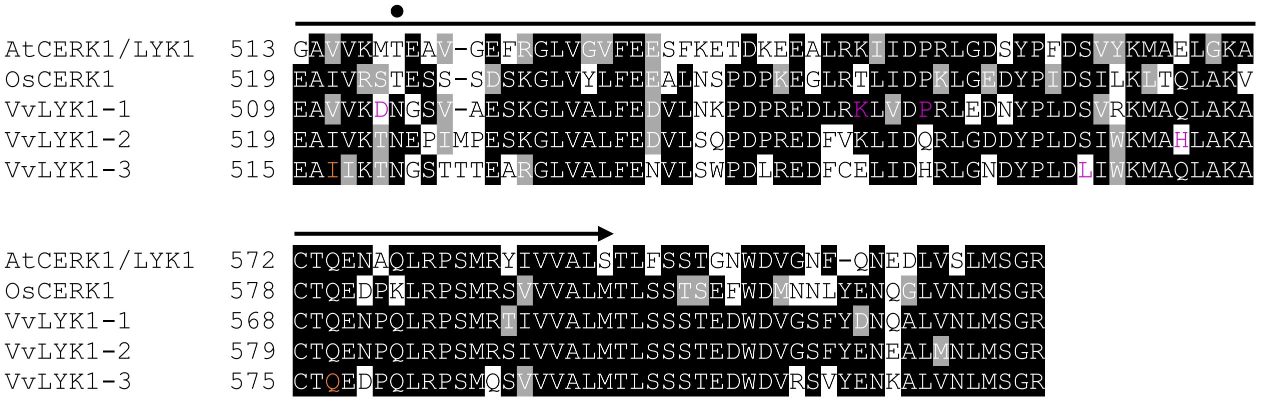

Fig. S3. Alignment of AtCERK1/LYK1, the rice OsCERK1 and its putative orthologs in grapevine (VvLYK1-1/-2/-3). Protein sequences were aligned with T-Coffee. Black and gray shading representing identical and positive amino acids, respectively, was visualized with Boxshade. The predicted signal peptide, the lysin motifs (LysM), the transmembrane region and the serine/threonine (S/T) kinase are shown. The residues of the chitin-binding site in AtCERK1/LYK1 LysM2 are indicated by asterisks and their conservation is highlighted in the grades of green. The residues E110, E114 and I141 bind to N-acetyl moieties of $(GlcNAc)_5$, while Q109, T112, Y113, A138, T139, N140, P142 and L143 interact with hydroxyl and hydroxymethyl groups of glucose part (Liu *et al.*, 2012). Conserved Cys residues (in red) form disulfide bridges (indicated by arrows). The RD type of kinase is highlighted in blue. SNPs between the NCBI reference sequence from *V. vinifera* cv Pinot Noir (PN40024) and cv Cabernet-Sauvignon are highlighted in purple (VvLYK1-1: Q99H, L220I, A221S, H264Q, S265T, S313T, D514E, K543R, P547A; VvLYK1-2: K67T, E73K, G95R, I186V, E216G, R279G, H574Q; VvLYK1-3: F116Y, I142L, V250A, A493E, L564F) or in orange when compared to cv Gamay (VvLYK1-1: no SNP; VvLYK1-2: no SNP; VvLYK1-3: -224A, V250A, A493E, I517V, Q577L). Residues S266, S268, S270, S274 and T519 (indicated by ●) were found to be phosphorylated in AtCERK1/LYK1 after chitin treatment (Petutschnig *et al.*, 2010). A: Ala, C:Cys, D:Asp, E:Glu, F:Phe, G:Gly, H:His, I:Ile, K:Lys, L:Leu, M:Met, N:Asn, P:Pro, Q:Gln, R:Arg, S:Ser, T:Thr, V:Val, W:Trp, Y:Tyr.

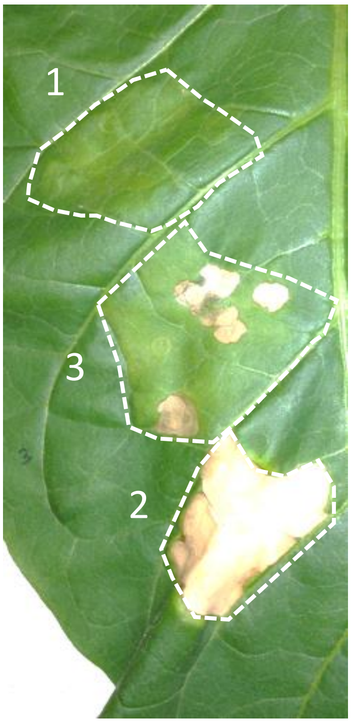

Fig. S4. Necrosis observed in response to the over-expression of *VvLYK1-2* in *Nicotiana benthamiana*. Symptoms observed 2d after leaf infiltration with (1) *A. tumefaciens* GV3101 alone (negative control), (2) *A. tumefaciens* GV3101 containing the construct *RPV1*$^{TIR1\text{-}193}$ (positive control; Williams et al. 2016), (3) *A. tumefaciens* GV3101 containing the construct *pART27-VvLYK1-2.*

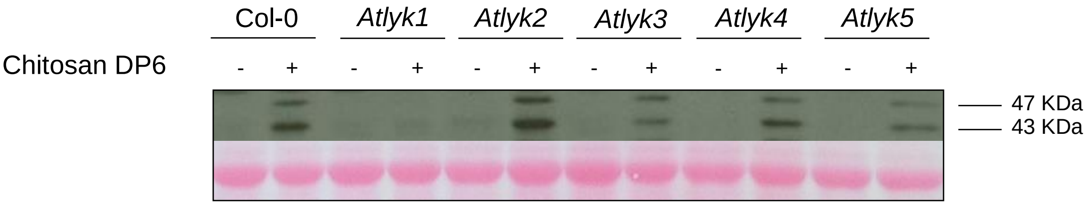

Fig. S5. Immunodetection of MAPKs in *Arabidopsis* mutants *Atlyk1-5* in response to chitosan. Plants of the different Arabidopsis *Atlyk* mutants have been treated with 1 $mg.mL^{-1}$ chitosan DP6 for 10 min before protein extraction. Activation of the two MAPKs was detected by immunoblot with an antibody raised against the human phosphorylated extracellular regulated protein kinase 1/2 (α-pERK1/2). Fifteen µg of proteins were loaded in each lane. Homogeneous loading was checked by Ponceau red staining. Similar results were obtained in three independent experiments.

Supplemental Tables

Table S1. The *Vitis vinifera* VvLYK family contains 15 putative genes in the grapevine genome.

NCBI Gene	NCBI mRNA	NCBI Protein	Previous annotation (Zhang et al. 2009)	Closest Arabidopsis homolog	Suggested new gene name	Gene length[1] (bp)	Expression validated with EST data	Expression validated with RNA-Seq data
LOC100255092	XM_010658923.2	XP_010657225.1	*VvLYK1*	*AtLYK1*	*VvLYK1-1*	1 845	Y	Y
LOC100257218	XM_010657064.2	XP_010655366.1	*VvLYK2*	*AtLYK1*	*VvLYK1-2*	1 878	Y	Y
LOC100262323	XM_010657063.2	XP_010655365.1	*VvLYK3*	*AtLYK1*	*VvLYK1-3*	1 866	Y	Y
LOC100264694	XM_019225274.1	XP_019080819.1	*VvLYK11*	*AtLYK2*	*VvLYK2*	2 028	Y	Y
LOC100256626	XM_002283592.4	XP_002283628.2	*VvLYK12*	*AtLYK3*	*VvLYK3-1*	1 815	Y	Y
**LOC100250732**	**XM_019219283.1**	**XP_019074828.1**	**Not described**	***AtLYK3***	***VvLYK3-2***	**Unknown**	**No ESTs**	**Incomplete coverage**
**LOC100256178**	**XM_002272778.4**	**XP_002272814.3**	**Not described**	***AtLYK3***	***VvLYK3-3***	**Unknown**	**2 ESTs**	**Incomplete coverage**
LOC100242712	XM_002269372.3	XP_002269408.1	*VvLYK4*	*AtLYK4*	*VvLYK4-1*	2 037	1 EST	Y
LOC100264999	XM_010650900.2	XP_010649202.1	*VvLYK6*	*AtLYK4*	*VvLYK4-2*	1 947	1 EST	Y
LOC100258108	XM_002277295.4	XP_002277331.3	*VvLYK10*	*AtLYK5*	*VvLYK5-1*	2 167	Y	Y
Not annotated: tBLASTn match on emb AM480420.1	MF939897	New sequence	Not described	*AtLYK5*	*VvLYK5-2*	2 078	Y	Y
LOC100264758	XM_002280034.2	XP_002280070.1	*VvLYK7*	No match	*VVLYK6*	2 200	No ESTs	Y
**LOC100259809**	**XM_002269436.4**	**XP_002269472.2**	***VvLYK5***	**No match**	***VvLYK7***	**Unknown**	**No ESTs**	**Incomplete coverage**
**LOC100248852**	**XM_002281844.3**	**XP_002281880.2**	***VvLYK8***	**No match**	***VvLYK8***	**Unknown**	**1 EST**	**Incomplete coverage**
**LOC100248488**	**XM_002276794.3**	**XP_002276830.3**	***VvLYK9***	**No match**	***VvLYK9***	**Unknown**	**2 ESTs**	**Incomplete coverage**

[1] Gene length based on Cabernet Sauvignon sequence - verified by RNA Seq data. Genes in bold = putative pseudogene

Table S2. Percentage of amino acid identity or similarity between VvLYK1-1/-2/-3 and AtCERK1/LYK1 or OsCERK1.

	whole protein sequence		LysM domain		Kinase domain	
	Identity	Similarity	Identity	Similarity	Identity	Similarity
**VvLYK1-1 vs AtCERK1**	60%	73%	46%	68%	77%	85%
**VvLYK1-2 vs AtCERK1**	57%	71%	44%	65%	74%	84%
**VvLYK1-3 vs AtCERK1**	56%	69%	51%	68%	70%	80%
**VvLYK1-1 vs OsCERK1**	61%	73%	44%	58%	80%	91%
**VvLYK1-2 vs OsCERK1**	58%	71%	42%	57%	79%	89%
**VvLYK1-3 vs OsCERK1**	56%	70%	43%	61%	74%	86%

Results were obtained with the NCBI protein BLAST program on the whole protein sequence or the LysM ectodomain or the kinase domain. Protein sequences used: AtCERK1/LYK1 (NP_566689), OsCERK1 (A0A0P0XII1), VvLYK1-1 (XP_010657225), VvLYK1-2 (XP_010655366), VvLYK1-3 (XP_010655365). Lys Motifs (LysM, PF01476) and Kinase domain (PF07714) were annotated with SMART. The signal peptide and the outer juxtamembrane region were not included in each LysM domain.

Table S3. Primers used in this study. For each primer, gene family, name used (ID), sequence (5'-> 3') and usage are reported. Underlined cacc is used for Gateway directional TOPO cloning. Letters in bold indicate restriction sites added to the 5' end of gene-specific primer sequences to facilitate cloning.

Gene Family	ID	Sequence (5'-3')	Usage
VvLYK1-1	*VvLYK1-1_RT_F*	tacatgccaccagaatacgc	Semi-quantitative RT-PCR
	VvLYK1-1_RT_R	aaggtcctctcgtggatcag	Semi-quantitative RT-PCR
	VvLYK1-1_R2	taaacagatccaaagccacc	Genotyping
	VvLYK1-1_F2	gtgggcttgtttacattcctac	Genotyping
	VvLYK1-1_qF	tggctttgttcgaggatgtg	qPCR
	VvLYK1-1_qR	cgagtgggtagttatcttcaagc	qPCR
	VvLYK1-1_Xho_F	cgc**ctcgag**caaatgaaacagaaggtggg	Vector construction
	VvLYK1-1_Xba_R	cgc**tctaga**aaggaaaaatggtgaagtattg	Vector construction
	VvLYK1-1_GW_F	caccatgaaacagaaggtgggtttaggg	Gateway construction
	VvLYK1-1_GW_R	ggcccttccagacattagattgacgagg	Gateway construction
VvLYK1-2	*VvLYK1-2_RT_F*	cgtcttgtgggtacatttgg	Semi-quantitative RT-PCR
	VvLYK1-2_RT_R	tcctcaaacaaagcaacgag	Semi-quantitative RT-PCR
	VvLYK1-2_F2	cccgaatttgtaaaaggaag	Genotyping
	VvLYK1-2_R2	cacaggaactgtatgctcatg	Genotyping
	VvLYK1-2_qF	gaccagaggcttggagatga	qPCR
	VvLYK1-2_qR	catcccaatcttcggttgatga	qPCR
	VvLYK1-2_Xba_R	cgc**tctaga**tgctaccttcctgacattag	Vector construction
	VvLYK1-2_Xho_F	ggc**ctcgag**aaacatggtcatttcatcaac	Vector construction
	VvLYK1-2_GW_F	caccatggtcatttcatcaaacagcaggaacgc	Gateway construction
	VvLYK1-2_GW_R	ggcccttcctgacattagattcatcagagcc	Gateway construction
VvLYK1-3	*VvLYK1-3_RT_F*	ttcttcattgcccactcgtc	Semi-quantitative RT-PCR
	VvLYK1-3_RT_R	caagtcctcttgcttcagtgg	Semi-quantitative RT-PCR
	VvLYK1-3_R2	atatcctatcaggcgaaccag	Genotyping
	VvLYK1-3_F2	gttgatttcagccttggtagtg	Genotyping
	VvLYK1-3_qF	ggcaatgactacccacttgac	qPCR
	VvLYK1-3_qR	ttagtgcgacaactactgactg	qPCR
	VvLYK1-3_Xba_R	cgc**tctaga**cacactatcttcctgacatc	Vector construction
	VvLYK1-3_Xho_F	gcg**ctcgag**ccatctttgctagggtttc	Vector construction
	VvLYK1-3_GW_F	caccatgttggtttttagaatctcaagg	Gateway construction
	VvLYK1-3_GW_R	ggctcttcctgacatcagattcactagagc	Gateway construction
Housekeeping genes	*AtEF1_F*	tgagcacgctcttcttgctttca	Semi-quantitative RT-PCR
	AtEF1_R	ggtggtggcatccatcttgttaca	Semi-quantitative RT-PCR
	AtOLI_qF	gagctgaagtggcttccatga	qPCR
	AtOLI_qR	cgtccgacatacccatgatcc	qPCR
	VvEF1α_qF	gaactgggtgcttgataggc	qPCR
	VvEF1α_qR	aaccaaaatatccggagtaaaaga	qPCR
Defense genes	*AtFRK1_qF*	tgaaggaagcggtcagattt	qPCR
	AtFRK1_qR	ctgactcatcgttggcctct	qPCR
	VvCHIT4C_qF	gcaaccgatgttgacatatca	qPCR
	VvCHIT4C_qR	cgtcgccctagcaagtgag	qPCR
	VvSTS1.2_qF	aggaagcagcattgaaggctc	qPCR
	VvSTS1.2_qR	tgcaccaggcatttctacacc	qPCR
	VvPAL_qF	agtctccatggacaacacccg	qPCR
	VvPAL_qR	tgctcagcactttcgacatgg	qPCR
	VvRBOHD_qF	caccaccatgcttcagtccctccat	qPCR
	VvRBOHD_qR	agcgatcttcttgaagacttgtcgcc	qPCR
